# Impact of the host immune response on the development of equine herpesvirus myeloencephalopathy in horses

**DOI:** 10.1099/jgv.0.001987

**Published:** 2024-05-20

**Authors:** K. S. Giessler, L. S. Goehring, S. I. Jacob, Allison Davis, M. M. Esser, Y. Lee, L. M. Zarski, P. S. D. Weber, G. S. Hussey

**Affiliations:** 1 Department of Pathobiology & Diagnostic Investigation, College of Veterinary Medicine, Michigan State University, East Lansing, MI, USA; 2 Bavarian Health and Food Safety Authority, Oberschleissheim, Germany; 3 MH Gluck Equine Research Center, College of Agriculture, Food & Environment, University of Kentucky, Lexington, KY, USA; 4 Pathology Core, Unit for Laboratory Animal Medicine, University of Michigan, Ann Arbor, MI, USA; 5 Department of Large Animal Clinical Sciences, College of Veterinary Medicine, Michigan State University, East Lansing, MI, USA

**Keywords:** equine herpesvirus 1 (EHV-1), equine herpesvirus myeloencephalopathy (EHM), herpesvirus pathogenesis, host immune response, IgG subisotype, interferon

## Abstract

Herpesviruses establish a well-adapted balance with their host’s immune system. Despite this co-evolutionary balance, infections can lead to severe disease including neurological disorders in their natural host. In horses, equine herpesvirus 1 (EHV-1) causes respiratory disease, abortions, neonatal foal death and myeloencephalopathy (EHM) in ~10 % of acute infections worldwide. Many aspects of EHM pathogenesis and protection from EHM are still poorly understood. However, it has been shown that the incidence of EHM increases to >70 % in female horses >20 years of age. In this study we used old mares as an experimental equine EHV-1 model of EHM to identify host-specific factors contributing to EHM. Following experimental infection with the neuropathogenic strain EHV-1 Ab4, old mares and yearling horses were studied for 21 days post-infection. Nasal viral shedding and cell-associated viremia were assessed by quantitative PCR. Cytokine/chemokine responses were evaluated in nasal secretions and cerebrospinal fluid (CSF) by Luminex assay and in whole blood by quantitative real-time PCR. EHV-1-specific IgG sub-isotype responses were measured by ELISA. All young horses developed respiratory disease and a bi-phasic fever post-infection, but only 1/9 horses exhibited ataxia. In contrast, respiratory disease was absent in old mares, but all old mares developed EHM that resulted in euthanasia in 6/9 old mares. Old mares also presented significantly decreased nasal viral shedding but higher viremia coinciding with a single fever peak at the onset of viremia. According to clinical disease manifestation, horses were sorted into an EHM group (nine old horses and one young horse) and a non-EHM group (eight young horses) for assessment of host immune responses. Non-EHM horses showed an early upregulation of IFN-α (nasal secretions), IRF7/IRF9, IL-1β, CXCL10 and TBET (blood) in addition to an IFN-γ upregulation during viremia (blood). In contrast, IFN-α levels in nasal secretions of EHM horses were low and peak levels of IRF7, IRF9, CXCL10 and TGF-β (blood) coincided with viremia. Moreover, EHM horses showed significantly higher IL-10 levels in nasal secretions, peripheral blood mononuclear cells and CSF and higher serum IgG3/5 antibody titres compared to non-EHM horses. These results suggest that protection from EHM depends on timely induction of type 1 IFN and upregulation cytokines and chemokines that are representative of cellular immunity. In contrast, induction of regulatory or TH-2 type immunity appeared to correlate with an increased risk for EHM. It is likely that future vaccine development for protection from EHM must target shifting this ‘at-risk’ immunophenotype.

**Impact statement**

Equine herpesvirus-1 (EHV-1) infection results in sporadic but devastating outbreaks of neurological disease in the equine population caused by a myeloencephalopathy with a poorly understood pathogenesis. Like alpha herpesviruses of humans and other animals, the impact of EHV-1 on health and the equine economy is significant. Despite the importance of EHV-1 in horses, effective prevention remains elusive and there is currently no vaccine available to prevent EHM. This is in large part a consequence of the absence of a reliable experimental equine model of EHM. The work described here demonstrates a neurological equine model to reliably induce EHM and uncovers the immunological basis of protection from EHM. We find that the protection from EHM is associated with timely induction of type 1 IFN and upregulation of cytokines and chemokines that are representative of cellular immunity. This knowledge is critical for targeted development of vaccines, adjuvants and therapeutics and provides a model for testing of such modalities. Furthermore, information on how host factors critically determine viral pathogenesis is relevant for alpha herpesviruses of other species.

## Data Summary

The authors confirm all supporting data, code and protocols have been provided within the article or through supplementary data files.

## Introduction

Herpesviruses have co-evolved with their host species by developing strategies to live in balance with their specific host’s immune system [1]. The International Committee on Taxonomy of Viruses classifies herpesviruses within the order *Herpesvirales* into three families: *Orthoherpesviridae* (former *Herpesviridae,* affecting mammals, avian and reptilian species), *Alloherpesviridae* (affecting fish) and *Malacoherpesviridae* (affecting molluscs) [2]. Within the family *Orthoherpesviridae,* nine herpesviruses have been identified that affect equids [3]. The clinically and economically most important equine herpesviruses (EHV) are EHV-1 and EHV-4, which are closely related representatives of the genus *Varicellovirus* and classified to the subfamily *Alphaherpesvirinae* [1,3]. Horses become infected with EHV-1 and EHV-4 early in life and these viruses are enzootic pathogens circulating worldwide within the horse population [4]. Clinical disease of both viruses is often limited to respiratory disease and fever in younger horses, and results in a life-long latent infection in many cases. This is the potential source for new disease outbreaks upon viral reactivation after a period of stress. However, in contrast to EHV-4 infections, EHV-1 infections can cause severe secondary disease manifestations. These include abortions, neonatal foal death, chorioretinopathy and equine herpesvirus myeloencephalopathy (EHM) [5,6].

Following infection with EHV-1, up to 10 % of infected horses develop EHM with neurological symptoms ranging from mild ataxia and urinary incontinence to complete paralysis and recumbency [6]. These EHM outbreaks have significant emotional and economic impact worldwide due to quarantine measures, travel restrictions and shutdowns of international events. Most recently, this was highlighted by a severe outbreak at an international show jumping event in Valencia, Spain, which resulted in the death of 18 horses and in related cases of infections across ten countries in Europe [7,8]. The Valencia outbreak lines up with other tragic examples of yearly EHV-1 outbreak series during recent years throughout Europe and the USA [9,12].

Despite the significant impact of EHM outbreaks, prevention is limited to biosecurity and quarantine regimes as currently available vaccines are not licensed to prevent EHM [13,14]. Because neurological disease remains sporadic and EHM is difficult to reproduce experimentally, evaluating EHM pathogenesis and developing EHM vaccines in the natural host is challenging [15,16]. This is highlighted by the fact that despite extensive research there is still a lack of knowledge about host factors that predict risk/protection from EHM and that can be used for vaccine development [17].

What is known is that EHV-1 infects the epithelial cells of the upper respiratory tract, followed by viral replication and intercellular spread, which coincides with the onset of respiratory disease and viral nasal shedding by 24–48 h post-infection (hpi) [6]. Subsequently, infectious virus is taken up by local lymphocytes and transported to the local lymphoid tissues, where it can be detected as early as 12–48 hpi [18,19]. A peripheral blood mononuclear cell (PBMC)-associated viremia is then established, which is essential for the viral transport to the vascular endothelium of secondary infection sites such as central nervous system (CNS), uterus, gonads or the eye [18,20,25]. This cell-associated viremia is typically detected between days 4 and 14 post-infection and corresponds with a secondary fever peak [14,26,28]. Once the virus has reached the vascular endothelium at secondary infection sites, subsequent infection and replication causes vasculitis and thrombosis [13,21]. Consequently, hypoxia and ischaemia results in necrosis of adjacent neural tissues, which leads to EHM and is termed as ‘equine-stroke’ [21]. Likewise, similar infection patterns in the endometrium of the pregnant uterus are thought to cause late term abortion [15,29, 30].

Infection of the CNS or the uterus is dependent on viremia, and a higher magnitude and longer duration of viremia is thought to correspond with an increased risk of EHM development [31,33]. Furthermore, DNA polymerase (ORF 30) strain variants characterized by a single nucleotide polymorphism resulting in an amino acid variation at position 752 to aspartic acid (D752) have been associated with high neuropathogenic potential and have been shown to be more effective in establishing higher viral loads and a prolonged duration of viremia than low-neuropathogenic strain variants [31,33]. However, low-neuropathogenic strains (N752) also have the potential to result in devastating EHM outbreaks, which was demonstrated during the Valencia outbreak in 2021 [7]. Other essential viral factors as well as host and environmental factors all contribute to the incidence of EHM in infected horses [11,34, 35].

Host factors that contribute to an increased risk for EHM include certain breeds such as warmblood horses, Fjord horses [36], the Draft horse, Standardbred and Hispanic breeds [35]. It has also been shown that the risk of developing EHM in aged female horses significantly increases to 70 % in mares >20 years of age, while younger horses tend to show more severe respiratory symptoms [32,35, 37, 38]. Age-related changes of the immune system are likely to increase the susceptibility to infectious diseases in geriatric horses similarly to what is known for the elderly human population [39]. Similarly, alterations of the immune response locally at the pregnant uterus are reported during late pregnancy due to hormonal changes and likely contribute to the pathogenesis of EHV-1 abortions [40,41]. Ultimately, the impact of a genetic component in high-risk breeds as well as the impact of age and sex on EHM development is not fully understood and further defining how these factors influence the immunological state and EHM is an important step in the prevention of EHM [11].

While there are several studies that have increased our understanding of immunity to EHV-1 [33,42,49], most of this information is based on *in vitro* models including primary tissue culture assays to simulate EHV-1 infection at the different anatomical sites [50]. However, ultimately these data must be related to the complex processes and cellular interactions during *in vivo* EHV-1 and EHM pathogenesis. To accomplish this, the goal of the current study was to define immune parameters that are associated with clinical EHM in an equine model, by using the ‘old mare model’ to experimentally induce clinical EHM.

## Methods

### Animals

A total of 18 mixed breed horses were selected for this study. The horses ranged in age from 2 to 21 years and were assigned to two experimental groups: the young horse group consisted of 2-year-old horses (*n*=9; six males, three females) and the group of aged female horses consisted of nine 18–21-year-old mares. All horses were tested for recent prior exposure to EHV-1 by assessing virus neutralization antibody titres. Young horses showed pre-challenge titres <8 and the aged horses exhibited pre-challenge titres <32. Animals were housed in a building with natural ventilation with multiple horses per pen and nose-to-nose contact between pens. Horses had access to grass hay and water *ad libitum* for the entirety of the study. All animal maintenance and procedures were performed in compliance with Michigan State University’s Institutional Animal Care and Use Committee, under protocol ‘PROTO201800015’.

### Experimental design and sample collection

The experimental design for this study was adapted from a previous study by Holz *et al*. [33] (Table 1). Viral challenge infections for each group were conducted 1 year apart. Both groups were infected by intranasal instillation of 5×10^7^ PFU. of EHV-1 Ab4 wild-type virus on day 0 of the study. All horses were then studied for 21 days unless euthanasia was required earlier due to severe neurological symptoms (Mayhew scale >2) in individual horses. Experimental procedures and sampling were performed at time points indicated in Table 1. Nasal swab samples were collected by using sterile polyester swabs (Puritan Medical Products), transferred into 1 ml of virus transport medium (PBS containing 0.5 % BSA, 2000 U ml^−1^ penicillin, 4 mg ml^−1^ streptomycin, 0.16 mg ml^−1^ gentamicin) and stored at −80 °C until further analysis. Blood samples were collected via jugular venipuncture into EDTA-vacutainer collection tubes (BD medical) for cell-associated viremia analysis, and PAXgene RNA system tubes (BD medical) for mRNA detection. For virus neutralization antibody (VN) titre and IgG isotype assessment, blood was collected into serum separator tubes (BD medical) and serum subsequently aliquoted and stored at −20 °C. Nasal secretion samples for local cytokine measurement were collected by placing a tampon into the ventral nasal meatus for 20 min as previously described [51]. The samples were then centrifuged at 2000 *g* and the nasal secretion fluid was aliquoted and stored at −20 °C until further analysis. For cerebrospinal fluid (CSF) sample collection, ultrasound-guided cervical centesis was performed between C1 and C2 as previously described [33,52]. CSF samples were instantly snap frozen in liquid nitrogen and stored at −80 °C.

**Table 1.** Experimental design

Day of study	−4	−3	CH	1	2	3	4	5	6	7	8	9	10	11	12	13	14	15	16		21
Physical exams	x	x		x	x	x	x	x	x	x	x	x	x	x	x	x	x		x	ad	x
Body temperatures	x			x	x	x	x	x	x	x	x	x	x	x	x	x	x		x	ad	x
Shedding	x	x		x	x	x	x	x	x	x	x	x	x	x	x	x	x		x	ad	x
Viremia	x			x	x	x	x	x	x	x	x	x	x								
EHV-1 VN and IgG Ab titres	x																x				x
Cytokines in nasal secretions	x			x	x																
Cytokines/chemokines in mRNA	x			x	x	x	x	x	x	x	x	x	x								
Cytokines in CSF		x												x							

ad, Sampling on alternate days; x, daily sampling; CH, challenge infection.

### Physical exams and clinical scores

Physical exams were performed on all horses prior to infection, daily until 14 days post-infection (dpi) and every other day until day 21 post-infection (pi) (Table 1). Respiratory disease evaluation included assessment of the presence and severity of ocular and nasal discharge and cough. The respective respiratory disease was graded on a scale from 0 (=normal) to 3 (moderate mucopurulent nasal and ocular discharge and ≥three episodes of coughing, respectively). A total respiratory score was established by adding up individual scores and each horse could get a respiratory disease score of up to 6 for each day (Table 2). Neurological symptoms were evaluated based on ataxia, dysmetria and weakness using an adaptation of the Mayhew ataxia scale as previously described [32,53, 54]. A daily neurological score was calculated for each horse (0=normal to 3=severe ataxia/recumbency, Table 2). In addition, rectal body temperature was taken twice daily until day 14 pi and a temperature ≥101.5 °F (38.6 °C) was considered as fever.

**Table 2.** Clinical scores

Clinical category	Clinical signs	Score
Ocular/nasal discharge	Normal, slightly serous	0
	Copius serous	1
	Slightly mucopurulent	2
	Moderately mucopurulent	3
Coughing	Normal	0
	Single episode	1
	Two episodes	2
	≥Three episodes	3
	**Daily respiratory disease score per horse**	**0–6**
CNS	Normal	0
	Reduced tail muscle tone	1
	Mild – moderate ataxia: Mayhew* ataxia grade 1–2	2
	Severe ataxia – recumbency: Mayhew* ataxia grade 3–5	3
	**Daily neurological disease score per horse**	**0–3**

*Mayhew *et al*. [53].

### Nasal viral shedding

Isolation of viral DNA from nasal swab samples was performed by magnetic bead-based DNA extraction using the IndiMag Pathogen Kit (Indical Bioscience) according to the manufacturer’s instructions. Subsequently, eluted DNA samples were quantified using a Nanodrop spectrophotometer (Thermo Fisher Scientific) and analysed by real-time quantitative PCR (qPCR) with primers and probe specific for the EHV-1 gB gene as previously described [26]. Briefly, each real-time PCR consisted of extracted template DNA, 12.5 µl TaqMan Fast Universal PCR Master Mix (2×), no AmpErase UNG (Applied Biosystems by Life Technologies), 400 nM forward and reverse primers, 200 nM probe and nuclease-free water added up to a total volume of 25 µl, and PCR was performed with a 7500 Fast Real-Time PCR System and 7500 Software v2.0.6 (Applied Biosystems by Life Technologies). For viral copy quantification, tenfold serial dilutions of EHV-1 gB plasmid DNA were included as a standard curve in each real-time PCR assay. Each sample was analysed in triplicate and the viral load was calculated as mean log EHV-1 gB copy number per 100 ng template DNA.

### Cell-associated viremia

Total DNA was isolated from anticoagulated blood samples and quantified with a Nanodrop spectrophotometer (Thermo Fisher Scientific). For analysing cell-associated viremia, the viral load was detected by qPCR using primers and probe specific for the EHV-1 gB gene as previously described [26] and each PCR was performed as described for nasal swab samples. Viral copy numbers were extrapolated to an EHV-1 gB plasmid standard curve as described above and normalized to 500 ng of template DNA.

### Determination of immunophenotypes associated with EHM or protection from EHM

Because one of the main goals for this study was to define the immunophenotype of horses with and without EHM, horses were sorted according to their clinical disease score into an EHM group (nine old horses, one young horse) and a non-EHM group (eight young horses) for cytokine analysis in nasal secretions and CSF, mRNA analysis in whole blood and the determination of antibody responses.

### Measurement of cytokines in nasal secretions and CSF

The analysis of cytokines and chemokines in nasal secretions and CSF included IFN-α, IFN-γ, IL-4, IL-17 and IL-10 by a fluorescent bead-based system (Luminex IS 100 instrument; Luminex) as previously described [33,55]. The data for IFN-α, IL-4 and IL-10 are presented in pg ml^–1^ and for IFN-γ and IL-17 in U ml^–1^.

### Measurement of cytokine and chemokine mRNA expression in blood

Total RNA was isolated from whole blood samples that were collected into PAXgene RNA Blood Tubes according to the manufacturer’s instructions (Qiagen). Following measurement of total RNA concentration with a Nanodrop spectrophotometer (Thermo Fisher Scientific), 20 ng µl^–1^ of sample RNA was used for reverse transcription with a High-Capacity cDNA Reverse Transcription Kit including RNase Inhibitor (Applied Biosystems). The template cDNA was analysed for cytokines/chemokines indicated in Table 3 by high-throughput qPCR using the SmartChip Real-Time PCR System (Takara Bio) (RT-qPCR) following the manufacturer’s instructions. Each reaction consisted of template cDNA, TaqMan Gene Expression Master Mix (Applied Biosystems) and TaqMan Gene Expression assays for equine target genes or in-house prepared primer/probe combinations as previously described [56]. Details on primers and probe design are given in Table 3. All samples were run in triplicate and no-template controls were included on each chip. Three housekeeping genes (GUSB, ACTB and YWHAZ) were included and averaged to normalize the expression of the respective gene of interest. The relative quantity (RQ) of each gene was analysed with the negative delta delta Cq (−ddCq) method as published by Livak and Schmittgen [57] using the average of the pre-challenge values for each group and for each gene assay as calibrators.

**Table 3.** Primer and probe source list

Gene	Source
Il-1β	TaqMan gene expression assay no.: Ec04260298_s1
IL-8	TaqMan gene expression assay no.: Ec03468860_m1
IL-10	TaqMan gene expression assay no.: Ec03468647_m1
IL-6	TaqMan gene expression assay no.: Ec03468678_m1
IL-12a	TaqMan gene expression assay no.: Ec03468747_m1
TGF-β	TaqMan gene expression assay no.: Ec03468030_m1
FoxP3	TaqMan gene expression assay no.: Ec04319948_m1
IL-2	TaqMan gene expression assay no.: Ec03468864_m1
IFN-α	Zarski *et al*. [56]
IFN-β	Young Go *et al*. [122]
CXCL10	TaqMan gene expression assay no.: Ec03469403_ml
CCL5	TaqMan gene expression assay no.: Ec03468106_m1
CCL3	TaqMan gene expression assay no.: Ec03469406_m1
THBSI	TaqMan gene expression assay no.: Ec04950867_g1
MMP9	TaqMan gene expression assay no.: Ec03469193_m1
IFN-γ	TaqMan gene expression assay no.: Ec03468606_m1
TBET	Ainsworth *et al*. [123]
IRF7	Oladunni *et al*. [124]
IRF9	Oladunni *et al*. [124]
B2M	TaqMan gene expression assay no.: Ec03468699_m1
GUSB	TaqMan gene expression assay no.: Ec03470630_m1
BACT	TaqMan gene expression assay no.: Ec04176172_gH
YWHAZ	TaqMan gene expression assay no.: Ec05951820_g1

### Virus neutralizing antibody testing

Virus neutralizing antibody titres in serum samples were analysed as previously described [33,58] with a few modifications. Briefly, each sample was heat inactivated for 30 min at 56 °C and serial dilutions from 1 : 4 to 1 : 4096 of each sample were established. Subsequently, serum dilutions were incubated with 500 TCID_50_ of EHV-1 in 96-well tissue culture plates (Corning) at 35 °C and 5 % CO_2_ for 1 h. Next, 4.5×10^5^ equine dermal cells (ATCC CCL-57) ml^–1^ were added and the plates were incubated at 35 °C and 5 % CO_2_ for 3 days before the assessment of cytopathic effect (CPE). The VN assay included a cell control and EHV-1-positive and EHV-1-negative serum control samples.

### IgG subisotype responses

Serum samples were also analysed in an IgG sub-isotype ELISA assay for specific IgG1, IgG4/7 and IgG3/5 antibody detection as previously described [58] with a few modifications. Briefly, 10 µg ml^−1^ of gradient-purified concentrated EHV-1 in carbonate-coating buffer was coated to 96-well polystyrene plates and incubated overnight at 4 °C. Following washing steps using PBS/0.5 % Tween (Thermo Fisher Scientific), plates were blocked with PBS/1 % fish gelatin (Sigma-Aldrich) and incubated for at least 1 h at room temperature. Subsequently, each of the samples diluted to 1 : 2000 (IgG1), 1 : 20 000 (IgG4/7) and 1 : 100 (IgG3/5) in 1 % fish gelatin (Sigma-Aldrich) blocking buffer was added to the plate in triplicate and incubated at room temperature for 2 h. In addition, a serially diluted serum sample of known titre was included on each plate to establish a four-parameter standard curve which was used to calculate the respective IgG sub-isotype titre of the serum samples. For detection of specific antibody sub-isotype responses to EHV-1, 1 : 10 diluted mAbs specific for equine IgG1 (CVS 45), IgG4/7 (CSV 39) and IgG3/5 (CVS 38) [59,60] were added following incubation of the samples/standards and a wash step. Following another wash step, plates were then incubated with peroxidase-conjugated Affinipure goat anti-mouse IgG and IgM (H+L) (Jackson Immuno Research Laboratories) at 1 : 2000 dilution. The colour was developed using 3,30,5,50-tetramethybenzidine substrate (Kirkegaard and Perry Laboratories) and the reaction was stopped using 1 M phosphoric acid followed by spectrophotometric measurement at 450 nm using an ELISA plate reader (Bio Tek Instruments).

### Statistics

For statistical analysis all data were tested for normality using GraphPad Prism Software v9. When possible, not normally distributed data were log transformed to achieve normal distribution. Data that were not normally distributed were analysed by non-parametric tests. Clinical scores and nasal shedding data were not normally distributed. Total respiratory and neurological scores were analysed by the Mann–Whitney test. For viral genome quantification assays in nasal swab samples, a multiple Mann–Whitney test with a false discovery rate approach and a two-stage step-up method of Benjamini, Krieger and Yekutieli (GraphPad Prism Software v9) was used. For viremia, the total duration and magnitude of viremia were compared using unpaired t-tests. In addition, the magnitude of viremia on each day viremia occurred was compared using unpaired t-test with a false discovery rate approach and a two-stage step-up method of Benjamini, Krieger and Yekutieli (GraphPad Prism Software v9). Similarly, body temperature data were normally distributed, and a multiple unpaired t-test using a false discovery rate approach with a two-stage step-up method of Benjamini, Krieger and Yekutieli (GraphPad Prism Software v9) was used. Because cytokine data in CSF and nasal secretions were not normally distributed, CSF cytokine data were analysed using a multiple Mann–Whitney test with a false discovery rate approach and a two-stage step-up method of Benjamini, Krieger and Yekutieli (GraphPad Prism Software v9). For nasal secretion cytokine data, a multiple Kolmogorov–Smirnov test with a false discovery rate approach and a two-stage step-up method of Benjamini, Krieger and Yekutieli (GraphPad Prism Software v9) was used to examine significance between the two experimental groups and a mixed-effects multiple comparison analysis with Dunnett’s correction was used to examine the effect of infection on cytokine levels. Antibody data were normally distributed. For VN and IgG subisotype antibody titres, EHM and non-EHM horses were compared by multiple unpaired t-tests using a false discovery rate approach with a two-stage step-up method of Benjamini, Krieger and Yekutieli (GraphPad Prism Software v9). The effect of infection on antibody titres was analysed using a mixed-effects multiple comparison analysis with Dunnett’s correction. Finally, the cytokine/chemokine mRNA gene expression data were normally distributed. For statistical analysis a multiple unpaired Holm–Šídák t-test comparison test was used to compare differences between EHM and non-EHM horses on each day. In addition, a multiple comparison two-way ANOVA with Dunnett’s correction was performed to evaluate cytokine mRNA expression on each day post-infection with pre-infection cytokine expression levels (GraphPad Prism Software v9). A *P*-value of <0.05 was regarded as a significant difference for all data.

## Results

### Old mares predominantly exhibited clinical EHM but no respiratory disease, while young horses showed respiratory disease following intranasal infection with EHV-1 Ab4, but only one young horse showed EHM

**Fig. 1.**
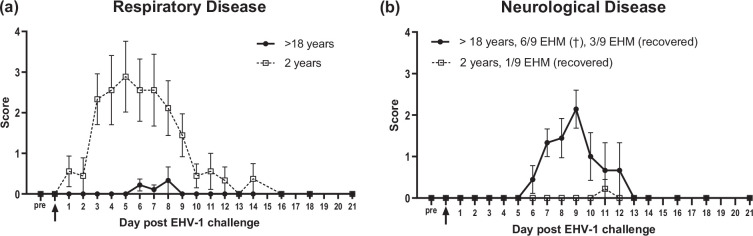
Clinical findings in old and young horses after intranasal infection with EHV-1 Ab4. (a) Respiratory disease score was calculated using individual scores for cough, nasal and ocular discharge. (b) Neurological disease score was determined by using the ataxia grading system established by Mayhew *et al*. [53,54] and Allen *et al*. [32]. Data are presented as mean values (±sem). Differences between groups were statistically significant (*P*=0.0001 respiratory score, *P*=0.004 neurological score).

The clinical disease evaluation included daily observations of respiratory and neurological symptoms. Clinical scores are presented as mean daily scores±sem in Fig. 1. Clinical disease outcomes differed significantly between the two infection groups. The young horse group showed significantly more respiratory symptoms throughout the study compared to old mares (*P*=0.0001) (Fig. 1a). Starting from day 1 pi, young horses developed ocular and nasal discharge, cough and pyrexia. Between day 3 and day 9 pi, respiratory symptoms were significant in young horses compared to pre-infection scores and compared to old horses, and the mean respiratory disease score peaked at day 5 pi. One horse continued to show considerable coughing until day 14 of the study. In contrast, none of the old mares exhibited respiratory symptoms for the first 5 days pi. While nasal and ocular discharge was absent in old mares throughout the study, a total of 3/9 horses showed mild single episodes of coughing on days 6 and 7 pi. and one horse coughed on day 8 pi.

In contrast, all old mares (9/9) developed neurological disease that resulted in euthanasia in 6/9 old mares due to severe EHM (Fig. 1b). This differed significantly (*P*=0.004) when compared to young horses, where a mild hind limb ataxia was observed in only 1/9 horses on day 11 pi. This hind limb weakness lasted for 1 day and the horse recovered fully afterwards. In old mares, the neurological symptoms started at day 6 pi. Within the following 3 days, severe ataxia developed in six old mares, three of which became recumbent, and all six horses were euthanized by day 9 pi due to severe EHM. Of the three old mares that survived, one horse showed mild to moderate ataxia starting on day 6 and lasting until day 12 of the study and recovered fully afterwards. The other two survivors showed reduced tail tone and mild ataxia for 1–2 days of the study but did not develop more severe symptoms.

**Fig. 2.**
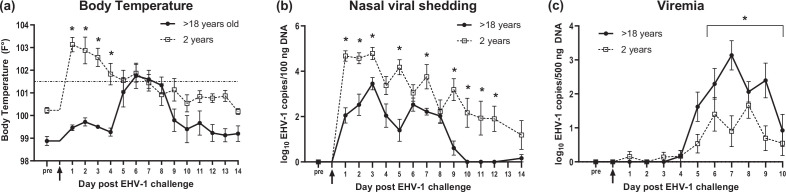
Body temperatures, nasal viral shedding and viremia in old and young horses after intranasal infection with EHV-1. (a) Mean body temperatures (°F). The dotted horizontal line represents the fever cut off at 101.5 °F. (b) Viral nasal shedding is expressed as log_10_EHV-1 gB copy number per 100 ng DNA detected in nasal swabs by qPCR. (c) viremia is shown as log_10_EHV-1 gB copy number per 500 ng DNA in PBMCs evaluated by qPCR. The data are presented as mean values (±sem). *Significant differences of viral load among groups (nasal viral shedding, viremia) or body temperature (*P*<0.05).

Body temperatures are shown in Fig. 2a and highlight the differences in clinical disease presentation between old mares and young horses. All young horses responded with a high primary fever starting on day 1 pi concurrently with the onset of respiratory symptoms (Fig. 1a) and high viral nasal shedding (Fig. 2b). Temperatures were significantly higher in young horses until day 4 pi (*P*<0.05) compared to old mares, which did not show a primary fever response. The initial high fever in young horses dropped on consecutive days but remained elevated until day 7 pi in most horses. Two young horses still showed a mild fever on day 13 pi. In contrast, the fever response of old mares was delayed and started on day 5 pi in three horses and 8/9 horses developed fever by day 8 of the study. The fever peaked in old mares on days 6 and 7 pi, which mirrored peak viremia (Fig. 2c) and the onset of neurological symptoms. The three old mares that survived showed normal body temperatures by day 9 pi and throughout the remaining days of the study. One of the old mares that survived did not develop fever during the whole study period.

### Young horses showed significantly higher viral nasal shedding, while old mares exhibited significantly higher viremia post-infection

Nasal viral shedding and cell-associated viremia are shown in Fig. 2b, c respectively. For nasal viral shedding, EHV-1 DNA was detected in all horses of both infection groups starting on day 1 pi except for one old mare, which started viral nasal shedding on day 2 pi. The young horse group showed significantly higher levels of nasal viral shedding on days 1–3 pi and days 5, 7 and 9–12 pi (*P*<0.05) when compared to the old mare group. Nasal viral shedding was overall reduced in old mares by day 9 pi but could still be detected in low levels by qPCR on day 14 pi in one individual. Three of nine young horses still showed low-level nasal viral shedding on the day of necropsy which was >day 20 pi (data not shown).

In contrast, while all horses developed some viremia, the overall duration and magnitude of viremia were significantly higher in old mares compared to the young horse group (*P*<0.05, Fig. 2c). Specifically, the overall average duration of viremia in old horses was 4.4 days compared to the young horses where overall average duration was 2.8 days. The magnitude of viral load was compared between groups by adding up the viral load of each day of viremia in the respective infection group. The average viral load peaked in old mares on the third day of viremia (days 6–10 pi), where it was 5486.4 EHV-1 copies per 500 ng DNA and significantly higher compared to the young horse group (173.2 EHV-1 copies per 500 ng DNA) (*P*<0.001). The day when viremia first occurred in each individual horse ranged between day 3 and 6 pi in both infection groups.

### Based on neurological scores, the EHM group contained all old horses and one young horse, and the non-EHM group contained eight young horses

Because one of the main goals for this study was to define the immunophenotype of horses with and without EHM, horses were sorted according to their clinical disease score into an EHM group (nine old, one young horse) and a non-EHM group (eight young horses) for the evaluation of cytokine responses in nasal secretions and the CSF, determination of mRNA analysis in whole blood and the determination of antibody responses.

### Protection from EHM was associated with significantly higher IFN-α and IL-17 responses in nasal secretions, but lower IL-10 secretion

**Fig. 3.**
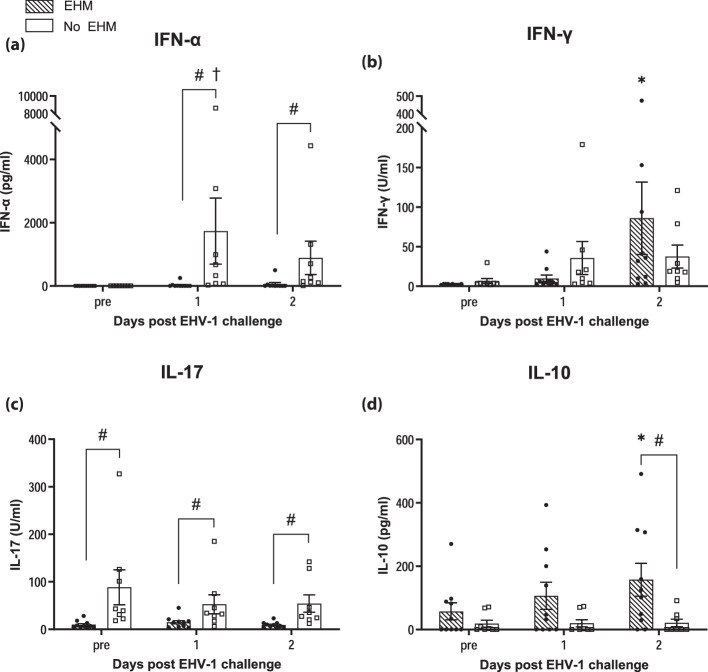
Cytokine levels in nasal secretions of EHM and non-EHM horses before and at days 1 and 2 post-experimental infection. IFN-α (**a**), IFN-γ (**b**), IL-17 (**c**) and IL-10 (**d**) were measured by Luminex and are indicated as concentration (pg ml^–1^). #Significant difference (*P*<0.05) comparing EHM and non-EHM horses. *,†Significant differences (*P*<0.05) compared to pre-infection values within each group.

Cytokine responses in nasal secretions were compared between horses exhibiting EHM (EHM group) and horses that did not develop EHM (non-EHM group). Average levels (pg ml^–1^) of IFN-α, IFN-γ, IL-17 and IL-10 (±sem) are presented in Fig. 3 (a–d). INF-α levels (Fig. 3a) increased in non-EHM horses and were significantly upregulated at 1 dpi compared to pre-infection values (*P*<0.05). In contrast, the IFN-α response in EHM horses was not significantly increased compared to pre-challenge levels. Moreover, EHM horses showed significantly lower IFN-α levels compared to non-EHM horses on days 1 and 2 pi (*P*<0.005).

INF-γ levels increased in both groups after infection and no significant difference could be detected between the two experimental groups (Fig. 3b).

No significant changes of IL-17 regulation were observed in response to infection in either group. However, non-EHM horses showed overall significantly higher IL-17 levels at all time points compared to EHM horses (*P*<0.05) (Fig. 3c).

In contrast, IL-10 levels were higher in EHM horses when compared to non-EHM horses at all time points measured (Fig. 3d). These increases in IL-10 levels were significantly upregulated compared to pre-infection values (*P*<0.005) in EHM horses and compared to IL-10 levels of non-EHM horses on day 2 pi (*P*<0.05).

### Cytokine/chemokine response associated with protection from EHM in whole blood was representative of a cellular immune response

**Fig. 4.**
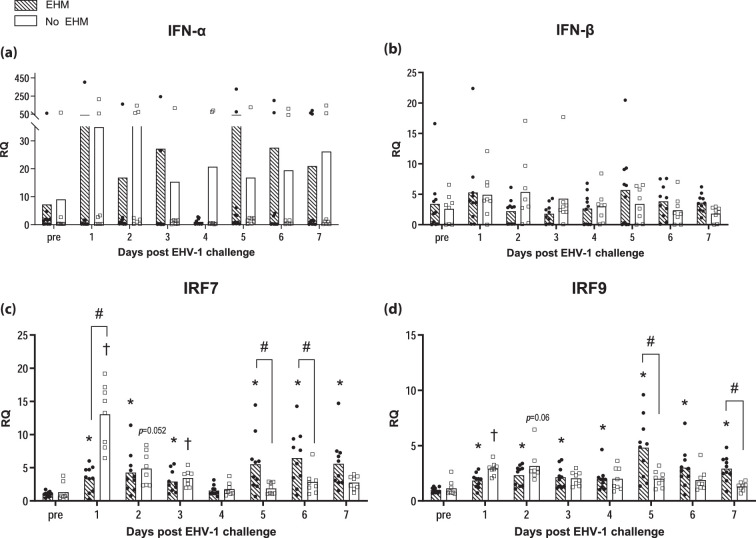
EHV-1-specific IFN response in PBMCs of EHM and non-EHM horses. Cytokine mRNA gene expression of IFN-α (**a**), IFN-β (**b**), IRF7 (**c**) and IRF9 (**d**). Relative quantities (RQ) represent *x*-fold increase over average levels observed for the same cytokine prior to experimental infection. #Significant difference (*P*<0.05) in post-hoc multiple comparison tests comparing different groups. *,†Significant differences (*P*<0.05) compared to pre-infection values.

Fold changes in the gene expression over the average pre-infection levels of selected genes were compared between EHM and non-EHM horses. RQ values for each gene are presented in Figs.4^6^.

**Fig. 5.**
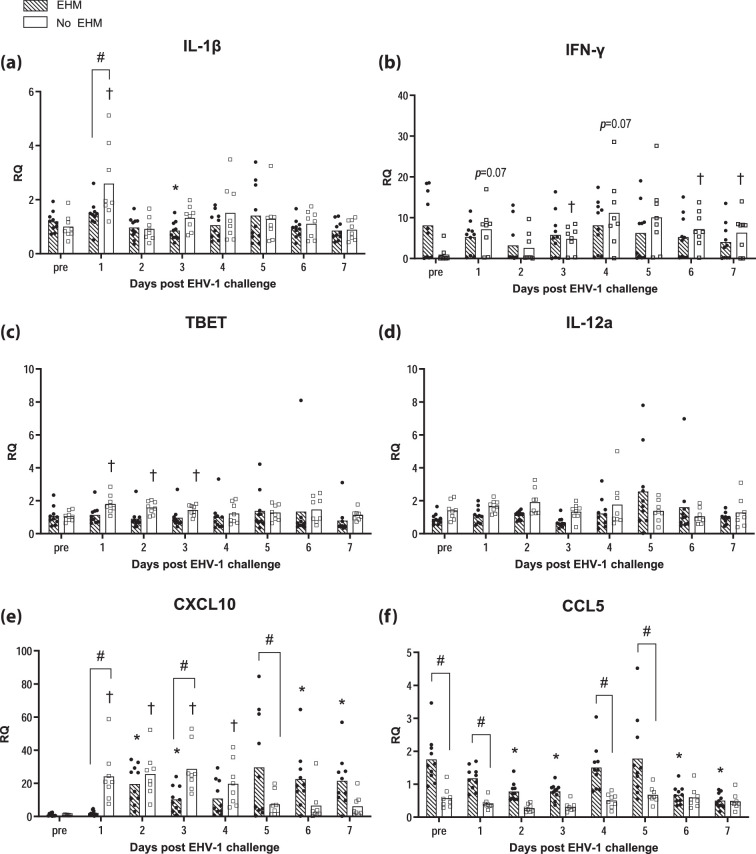
EHV-1-specific proinflammatory cytokine and chemokine response in PBMCs of EHM and non-EHM horses. Cytokine mRNA gene expression of IL-1β (**a**), IFN-γ (**b**), TBET (**c**), IL-12a (**d**) and chemokine mRNA gene expression of CXCL10 (**e**) and CCL5 (f). Relative quantities (RQ) represent *x*-fold increase over average levels observed for the same cytokine/chemokine prior to experimental infection. #Significant difference (*P*<0.05) in post-hoc multiple comparison tests comparing different groups. *,† Significant differences (*P*<0.05) compared to pre-infection values.

**Fig. 6.**
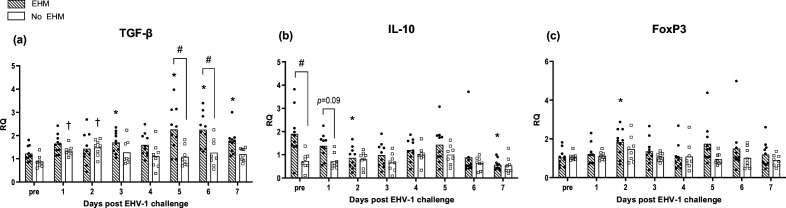
EHV-1-specific regulatory cytokine response in PBMCs of EHM and non-EHM horses. Cytokine mRNA gene expression of TGF-β (**a**), IL-10 (**b**) and FoxP3 (**c**). Relative quantities (RQ) represent *x*-fold increase over average levels observed for the same cytokine prior to experimental infection. #Significant difference (*P*<0.05) in post-hoc multiple comparison tests comparing different groups. *,†Significant differences (*P*<0.05) compared to pre-infection value.

### Protection from EHM was associated with an early IFN response in whole blood

No significant differences of IFN-α and IFN-β cytokine mRNA expression could be observed in whole blood in either group of horses. Differences in the timing of IFN pathway regulation became obvious between both groups when comparing the IFN regulatory gene expression of the interferon regulatory factor (IRF) 7 and IRF9 (Fig. 4).

Non-EHM horses responded with an early and significantly higher upregulation of IRF7 mRNA compared to EHM horses at day 1 after challenge (Fig. 4c). In EHM horses IRF7 and IRF9 mRNA levels did not change significantly until day 4 pi. By day 5 pi and coincidently with time-points of peak viremia, IRF7 and IRF9 mRNA levels increased significantly and exceeded levels of non-EHM horses (days 5 and 6 pi, IRF7; days 5 and 7 pi, IRF9).

### Protection from EHM was associated with induction of proinflammatory cytokine and IFN-γ responses in whole blood

Non-EHM horses responded with an early significant upregulation of the proinflammatory cytokine IL-1β (Fig. 5a). This IL-1β upregulation in non-EHM horses was significantly higher compared to EHM horses on day 1 pi.

Additionally, protection from EHM was associated with induction of cellular immunity including increased IFN-γ and TBET mRNA expression (Fig. 5b and c). IFN-γ mRNA expression was increased in non-EHM horses at day 1 pi (*P*=0.07), and these increases reached statistical significance during viremia at days 3, 6 and 7 pi. Overall, IFN-γ mRNA levels were significantly higher in non-EHM horses compared to EHM horses (Fig. 5b). Similarly, TBET mRNA expression was significantly increased in response to infection in non-EHM horses only (Fig. 5c). No significant induction of an IFN-γ response in response to infection could be observed in EHM horses. No significant induction of IL-12a gene expression was observed in either infection group (Fig. 5d).

### Chemokine responses mirrored pro-inflammatory phenotype observed in horses protected from EHM

Consistent with findings for IL-1β and TBET, the proinflammatory chemokine CXCL10 mRNA was upregulated post-infection in non-EHM horses (Fig. 5e), and this upregulation was significantly higher compared to EHM horses on days 1 and 3 pi.

In contrast, EHM horses showed a delayed upregulation of CXCL10, and levels peaked on days 5–7 pi, which mirrored the time of peak viremia.

For CCL5, significantly higher pre-existing and post-infection CCL5 levels were observed in EHM horses compared to non-EHM horses, which showed no significant response in terms of CCL5 gene expression at any time point (Fig. 5f). Interestingly, in EHM horses CCL5 levels were downregulated compared to pre-infection levels on days 2 and 3 pi, and on days 6 and 7 pi.

For CCL3, no significant changes in response to EHV-1 challenge could be observed for either infection group, although significantly higher levels of CCL3 were detected in EHM horses compared to non-EHM horses during peak viremia on day 7 pi (data not shown).

### EHM horses expressed increased levels of regulatory cytokine in whole blood

While EHM horses exhibited later induction of IFN responses and decreased induction of inflammatory and TH-1 type cytokine responses compared to non-EHM horses, we observed a significantly higher expression of regulatory cytokine mRNA levels (TGF-β and IL-10) (Fig. 6a, b). While non-EHM horses responded with an initial TGF-β upregulation at days 1 and 2 pi, this upregulation was not sustained after day 2 pi. In contrast EHM horses showed an upregulation of TGF-β mRNA levels in response to infection that was statistically significant on days 3 and 5–7 pi (*P*<0.05) and significantly exceeded TGF-β levels of non-EHM horses by days 5 and 6 pi (*P*<0.01). EHM horses also showed significantly higher pre-infection IL-10 cytokine mRNA levels compared to non-EHM horses (*P*<0.001). Following infection, IL-10 levels still exceeded levels of non-EHM horses on day 1 pi, although this trend did not reach significance (*P*=0.09) (Fig. 6b). IL-10 levels were downregulated in EHM horses compared to pre-infection values on days 2 and 7 pi. No significant changes in IL-10 levels were observed in non-EHM horses. FoxP3 mRNA expression was upregulated in EHM horses on day 2 pi, but no other significant changes could be detected for either experimental group throughout the study period (Fig. 6c).

### Thrombospondin 1 (THBS1) and matrix metalloprotease 9 (MMP9) gene expression fluctuate in the week following infection and are differentially regulated in whole blood of EHM and non-EHM horses

**Fig. 7.**
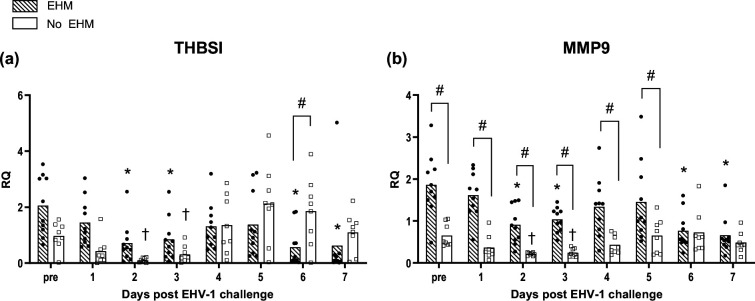
EHV-1-specific response of THBSI and MMP9 genes in PBMCs of EHM and non-EHM horses. mRNA gene expression of THBSI (**a**) and MMP9 (**b**). Relative quantities (RQ) represent *x*-fold increase over average levels observed for the same cytokine prior to experimental infection. #Significant difference (*P*<0.05) in post-hoc multiple comparison tests comparing different groups. *,†Significant differences (*P*<0.05) compared to pre-infection values.

Fold changes in the mRNA expression of genes involved in fibrinogen complex formation (THBS1) and in the pathogenesis of cerebral blood vessel damage (MMP9) are presented in Fig. 7. Both experimental groups responded with an initial downregulation of THBS1 expression on day 2 and 3 pi (*P*<0.05) (Fig. 7a). By day 6 pi, mRNA levels of non-EHM horses were significantly higher when compared to EHM horses, where mRNA expression was again downregulated compared to pre-existing values (days 6 and 7 pi, *P*<0.05).

EHM horses showed significantly higher levels of MMP9 mRNA pre-infection and from day 1 to day 5 pi when compared to non-EHM horses (*P*<0.05) (Fig. 7b). Overall, the MMP9 mRNA response was downregulated compared to pre-values in both groups at days 2 and 3 pi (*P*<0.05). In addition, MMP9 levels of EHM horses were then downregulated on days 6 and 7 pi (*P*<0.05) compared to pre-infection levels.

### Pre-existing IL-10 of EHM horses were elevated in the CSF of 7/9 horses, while IFN-γ, IL-17 and IL-4 levels were unremarkable

**Fig. 8.**
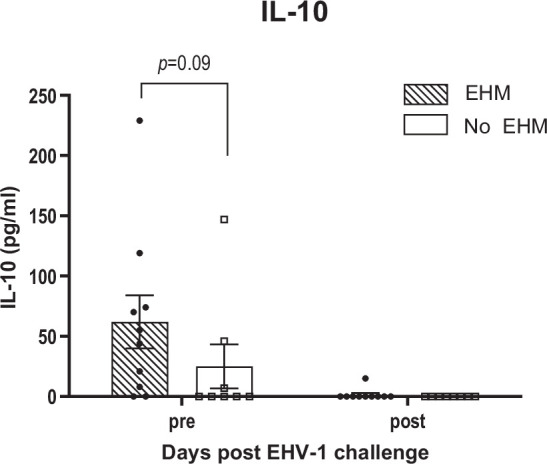
IL-10 levels in CSF of EHM and non-EHM horses. The IL-10 response was measured by Luminex and is indicated as concentration (pg ml^–1^).

Average levels of IL-10 in the CSF of EHM and non-EHM horses are shown in Fig. 8. Seven out of nine EHM horses showed higher pre-existing levels of IL-10 compared to non-EHM horses, but the average difference did not reach significance (*P*=0.09). Following EHV-1 infection, IL-10 secretion was inhibited in 8/9 EHM horses and completely absent in non-EHM horses.

Pre-infection IFN-α, IFN-γ, IL-17 and IL-4 levels were low or absent in both infection groups and no significant changes were observed post-infection (data not shown).

### Virus neutralizing antibody and IgG isotype responses

**Fig. 9.**
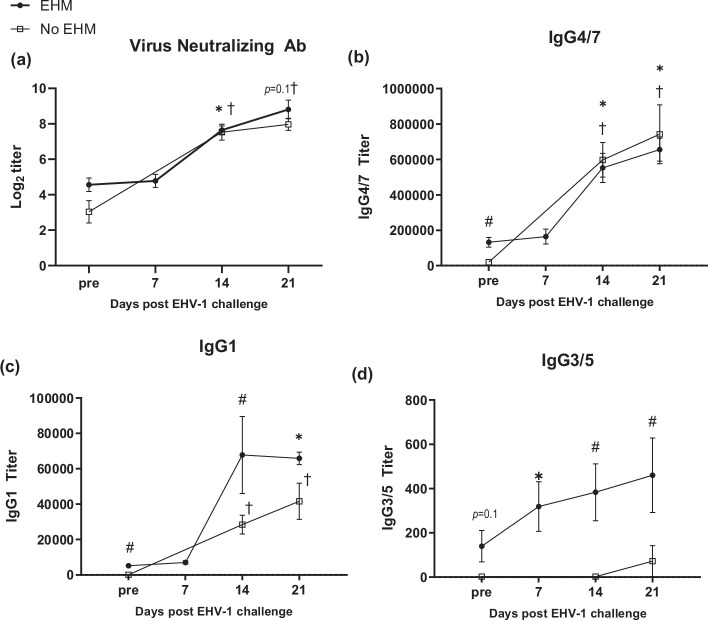
Virus neutralization antibody and IgG isotype responses of EHM and non-EHM horses. VN (**a**), IgG4/7 (**b**), IgG1 (**c**) and IgG3/5 (**d**) antibody titres. #Significant differences between groups. *,†Significant differences (*P*<0.05) compared to pre-infection values.

VN titres and IgG isotype responses of EHM and non-EHM horses are shown in Fig. 9. For EHM horses, an additional day of measurement was included on day 7 pi because 6/9 EHM horses had to be euthanized before the day 14 pi collection point. Thus, antibody levels for EHM horses on days 14 and 21 are represented by values of the three surviving EHM horses.

VN titres were low prior to infection and significantly increased in both groups starting by day 14 pi (Fig. 9a), but VN levels between the groups were not significantly different at any time point.

EHM horses had significantly higher IgG4/7 antibody levels prior to EHV-1 challenge compared to non-EHM horses (*P*<0.05) (Fig. 9b). Following EHV-1 challenge, the IgG4/7 response mirrored the VN response showing a significant increase in antibody levels by day 14 pi in both infection groups, but no significant differences were detected between experimental groups at any time point post-infection.

Pre-existing IgG1 antibody levels were also significantly higher in EHM-horses compared to non-EHM horses (*P*<0.001) (Fig. 9c). Following infection, IgG1 levels remained significantly higher for EHM horses at 14 dpi (*P*<0.05) and both infection groups showed a significant increase of IgG1 levels by day 21 pi (*P*<0.001).

IgG3/5 antibody levels prior to infection in EHM horses exceeded the concentration of non-EHM horses (*P*=0.1) (Fig. 9d). IgG3/5 levels increased in EHM horses following EHV-1 infection significantly by day 7 pi (*P*<0.05) and levels were significantly higher compared to non-EHM horses on days 14 (*P*<0.005) and 21 pi (*P*<0.05). Only small amounts of IgG3/5 were present in serum samples of non-EHM horses and levels did not change following EHV-1 challenge, except for one horse which showed a moderate increase by day 21 pi.

### EHV-1 risk evaluation on pre-infection sera

An adapted summary of the recommended interpretation of the EHV-1 risk assessment results is presented in Table 4 and the antibody results assessed using the Cornell ‘EHV-1 Risk evaluation assay’ are presented in Table 5a, b.

**Table 4.** EHV-1 risk evaluation – test interpretation

Risk evaluation	Total IgG (mean fluorescent intensity (MFI))		IgG4/7 (MFI)	Recommendation
High	<3000	&	<400	Immediate vaccination required
Moderate	≥3000	&	<400	Vaccination required within 1 month
	<3000	&	≥400	
Low	>3000	&	>400	Vaccination required within 3–6 months
Very low	>12 000	&	>10 000	Vaccination not needed for the next 12 months

Interpretation EHV-1 Risk Evaluation, table adapted from https://app.vet.cornell.edu/ahdc-portal/test-fee/details?test_code=EHV1PSP.

**Table 5.** (a) Pre-infection total IgG and IgG4/7 titres; (b) pre-infection total IgG and IgG4/7 titres for horses from Holz *et al*. [33]

Experimental group	Horse no.	PRE total IgG	PRE IgG4/7	Risk category	Neurological disease score	Respiratory disease score	
						o/n discharge	Coughing
**(a)**							
Y	27	236	2		0	3	3
Y	34	592	4		0	2	3
Y	41	656	4		0	3	3
Y	16	745	5		0	0	0
Y	28	1034	15	High	0	3	3
Y	35	1058	10		0	3	3
Y	23	1232	9		0	0	0
Y	26	1326	16		0	3	3
O	4	2634	79		3, †	0	1
O	1	2810	140		3, †	0	1
Y	15	4632	112	Moderate	2	0	0
O	9	4298	466		3, †	0	0
O	7	4823	425		3, †	0	0
O	6	4871	508		1	0	1
O	3	5285	578	Low	3, †	0	0
O	5	5477	926		3, †	0	0
O	2	7905	1888		2	0	3
O	10	11 891	6277		1	0	0
**(b)**							
Y2017	113	149	16		0	3	2
Y2017	110	155	14		0	3	1
Y2017	115	258	15		0	2	0
Y2017	121	1416	49	High	3, †	1	0
Y2017	122	1439	47		3, †	1	2
Y2017	112	1557	46		0	2	0
Y2017	120	2931	204		2	0	0

(a) o/n=ocular/nasal; †=euthanasia; Y=Young Horse Group (<2 years); O=Old Horse Group (>18 years). This table displays the pre-infection (PRE) total IgG antibody titres and IgG4/7 sub-isotype titres analysed in pre-serum samples of each horse by multiplex assay at the Animal Health Diagnostic Center, College of Veterinary Medicine, Cornell University. The risk category was assessed using the EHV-1 Risk Evaluation test interpretation that was provided (Table 4). The maximal neurological and respiratory disease score that was obtained during the present study for each respective horse is presented.

(b) o/n=ocular/nasal; †=euthanasia; Y2017=Young Horse Group (< 2 years) (Holz *et al*. 2017). This table displays the pre-infection (PRE) total IgG antibody titres and IgG4/7 sub-isotype titres analysed in pre-serum samples of each horse infected with EHV-1 Ab4 as part of the 2017 study by Holz *et al*. [33]. The risk category was assessed using EHV-1 Risk Evaluation test interpretation that was provided in Table 4. The maximal neurological and respiratory disease score that was obtained during the present study for each respective horse is presented.

For horses of the present study, the analysis of pre-serum samples categorized 10/18 horses to be high-risk (total IgG antibodies <3000 MFI and IgG4/7<400 MFI), 1/18 horse to be moderate-risk (total IgG antibodies >3000 MFI and IgG4/7<400 MFI) and 7/18 horses to be low- risk (total IgG antibodies >3000 MFI and IgG4/7>400 MFI) for EHV-1 infection.

For the high-risk category, 2/10 horses were representatives of the old horse group and developed ataxia (grade 4–5) resulting in euthanasia following EHV-1 challenge. Eight out of ten horses in the high-risk group were young horses and developed severe respiratory disease but did not show neurological symptoms. The one horse that was evaluated as a moderate risk candidate was a young horse and developed mild hind limb ataxia grade 1–2 and recovered fully afterwards. The seven horses of the low EHV-1 risk category were old horses. Following EHV-1 challenge infection, all seven horses developed neurological symptoms ranging from mild symptoms in three horses to severe ataxia resulting in euthanasia in four horses. The three horses with mild neurological symptoms, assessed as ataxia (grade 1–2) and weak tail tone, recovered fully, and survived.

An additional seven pre-serum samples of horses used in a separate previous study [33] were also tested using the EHV-1 Risk Evaluation assay. All seven horses showed total IgG antibody levels <3000 MFI and IgG4/7<400 MFI. This quantification identifies all seven horses as being high-risk for EHV-1 infection. During the study, 4/7 horses developed moderate–severe respiratory disease symptoms on multiple days of the study and did not show neurological symptoms. Moreover, 3/7 horses developed clinical EHM with two horses showing severe ataxia (grade 4–5) that resulted in euthanasia and one horse showing moderate hind limb ataxia (grade 1–2). One of the three EHM horses did not show any respiratory symptoms throughout the study period and the other two EHM horses did show mild respiratory symptoms on 1–3 days of the study.

## Discussion

The aim of this study was to identify the key elements of innate and adaptive host immune responses associated with development or protection of EHM following infection with EHV-1. Based on data previously described by Allen *et al*., which show the risk for developing clinical EHM following EHV-1 infection dramatically increases with age to >70 % [32], we used mares >18 years of age to experimentally induce EHM. Our hypothesis was that this ‘old horse model’ can be used to induce EHM and define the immunological phenotype associated with the increased risk from EHM. Our results validate the ‘old horse model’ as an experimental neurological model and show neurological symptoms in 9/9 old mares that were associated with a single fever spike at the onset of viremia in the absence of respiratory disease. In contrast, young horses showed a characteristic bi-phasic fever response following EHV-1 infection that correlated with the onset of respiratory disease and the onset of viremia. Interestingly, while all young horses developed significant respiratory disease, only 1/9 young horses developed mild–moderate neurological symptoms. Our results highlight the difficulties for vaccine development where young (presumably EHV-1 naive) horses are commonly used. Two recent systematic reviews of the literature show that commercial vaccines can reduce pyrexia, respiratory disease and nasal viral shedding [61,62]. However, there is no evidence that vaccination fully prevents viremia, no evidence that it prevents the occurrence of EHM, and there is limited evidence that killed vaccines reduce the incidence of abortion [10,11, 61,64]. This is perhaps not surprising when considering that vaccines are not tested in age groups where EHM occurs commonly. For the same reason, *in vivo* data on what type of immune response correlates with protection from EHM are lacking or incomplete at best.

In addition to showing different clinical manifestations, the magnitude of nasal viral shedding and viremia also differed significantly between experimental groups. Young horses showed significant nasal viral shedding from day 1 pi that coincided with respiratory symptoms and a high primary fever response followed by a secondary transient fever with the onset of viremia. These findings resemble the characteristic course of EHV-1 respiratory disease in young horses that has been comprehensively described in the literature [11,19, 26, 28, 33, 43, 44, 65,68]. In the absence of a primary fever and respiratory disease, the nasal viral shedding was significantly lower in old mares compared to young horses.

Viremia is a known prerequisite for EHM because it facilitates transport of EHV-1-infected PBMCs to the vascular endothelium of the CNS [6,28, 32]. It is thought that the magnitude and duration are associated with the likelihood of infection of the vascular endothelium of secondary infection sites and the risk for developing neurological disease [23,32]. Our results support this theory, as old mares showed a significantly higher magnitude of viremia compared to young horses and all old mares developed clinical EHM resulting in euthanasia of 6/9 horses. One of the old mares showed only a minimal transient fever during the study period. This horse also showed a low magnitude of viremia and was one of the horses that only developed mild ataxia. In contrast, only one of the young horses showed neurological symptoms and recovered without intervention. This emphasizes that the onset and magnitude of viremia are associated with secondary disease development. However, a recent systematic review found that while viremia is a prerequisite for secondary disease, there is limited current evidence that a reduction in the duration or magnitude of viremia will affect abortion or EHM outcomes [64]. While these results are probably affected by the limited availability of experimental data that include horses exhibiting EHM, they also highlight that additional host-associated factors are important in EHM pathogenesis and even horses with limited viremia can still be affected by EHM.

Consistent with previous reports [11,32], our study shows that old mares are more affected by EHM, they are less prone to respiratory disease and exhibit less viral nasal shedding. This apparent inverse correlation between the level of the respiratory response (or disease) and the occurrence of EHM is interesting and implies that the respiratory disease, including an initial fever and high nasal viral shedding, initiates a timely and strong innate immune response as a first line of defence [33,44, 65, 69]. Previously, EHV-1 viral nasal shedding has been shown to be correlated with levels and duration of IFN-α secretion [44,70]. In the present study we find that horses that were protected from EHM showed significantly increased INF-α levels in nasal secretions by 1 dpi compared to pre-challenge values and significantly higher induction of INF-α secretion compared to horses affected by EHM. In contrast, the INF-α response in nasal secretions of EHM horses was almost completely absent and did not change compared to pre-challenge levels. While most EHM horses in the present study were ‘old mares’, a previous study in yearling horses by Holz *et al*. also showed that following challenge infection with EHV-1 Ab4 IFN-α was reduced in nasal secretions of three horses that exhibited clinical EHM, when compared to the five age-matched horses that did not show any symptoms of EHM [33]. While it has been previously shown that induction of nasal IFN-α following experimental infection of horses with EHV-1/Ab4 is common in horses without EHM [33,43, 44, 65, 67, 69, 71, 72], and following EHV-1 infection of the respiratory epithelial cells *in vitro* [46,49, 73], the current study is the first to confirm an absence of nasal IFN induction in EHV-1-infected horses that go on to develop EHM. IFNs are cytokines that serve as ‘danger-signals’ in response to viral infections and induce an antiviral state in uninfected neighbouring cells through intracellular signalling cascades [74]. This leads to limitation of replication and viral spread [42,43, 69]. Particularly for neurovirulent EHV-1 strains, high IFN-α concentrations at the respiratory mucosa have been noted to be essential for the effective elimination of virus *in vitro* [46], but the mechanism by which nasal IFN-α contributes to EHM pathogenesis remains to be further elucidated *in vivo*.

In addition to the significant induction of an IFN-α response in non-EHM horses, we also observed higher levels of IL-17 in nasal secretions of horses protected from EHM compared to EHM horses. IFN-α and IL-17 are both signalling components of a TH-1 immune pathway [75]. This pathway is important in the activation of EHV-1-specific cytotoxic T-cells (CTLs), which are thought to be essential in the protection from EHM [32,76, 77].

Interestingly, while induction of nasal IFN-α and IL-17 as signalling components of a TH-1 immune pathway were significantly decreased in EHM horses, we detected significantly higher levels of the regulatory cytokine IL-10 in nasal secretions of EHM horses compared to non-EHM horses. Induction of IL-10 during the innate immune response is known to be an important feedback regulator that prevents immunopathology through inhibition of exaggerated immune reactivity [78]. We speculate that the high anti-inflammatory signals mediated through IL-10 pre- and post-challenge with a concurrently diminished mucosal IFN-α response may be indicative for a dysregulated innate immune response at the respiratory tract during EHV-1 infection in horses that go on to develop EHM. These changes in the innate immune response at the respiratory tract observed in EHM horses of the present study probably result in a local microenvironment that allows for the virus to spread efficiently through the connective tissue via infected mononuclear cells. In addition, the changes at the respiratory mucosa appear to lead to a failure of adequate priming of a protective adaptive immune response to combat the subsequent systemic viral spread during viremia. This is supported in the present study by the significantly increased viremia in EHM horses.

Further supportive of this theory is that we also observed a significantly delayed coordination of a timely type 1 IFN response in the blood of EHM horses of the present study. Induction of type 1 IFNs requires the activation and transcription of several IFN-stimulated genes (ISGs) [74]. This signalling network is orchestrated in mammals by transcription factors including IRFs, above all IRF7, which serves as the main regulator and predominantly induces IFN-α gene transcription [74,79]. Consistent with findings at the respiratory tract, activation of IRF7 and IRF9 mRNA expression was only significantly increased immediately following infection in non-EHM horses. In contrast, IRF7 mRNA expression was significantly lower in EHM horses on day 1 pi until the onset of viremia by day 5 pi when IRF7 and IRF9 mRNA levels were significantly higher in EHM horses when compared to non-EHM horses. Interestingly, while observing these differences at the level of gene regulation, no significant differences were detectable in IFN-α and IFN-β cytokine mRNA expression levels in the blood. A possible explanation for this discrepancy might be that type I IFNs are often transiently expressed, and levels can change within hours [80,81]. Thus, the analysis of once daily collected whole blood only provides a snapshot of the transient expression and quickly changing cytokine levels [82]. A further evaluation of regulation pathways may therefore be beneficial to understand the complex expression patterns during the immune response.

The local innate immune response is critical for priming and shaping activation of the adaptive immune system through cytokine and chemokine signalling. Upon activation, T helper cells can differentiate into TH-1, TH-2, TH-17 and regulatory T-cell (Treg) subsets [83]. An effective antiviral response to EHV-1 is thought to rely on the polarization towards TH-1 type cell-mediated immunity with the activation of CTLs [75,77,84], and high levels of precursor CTLs are thought to correlate with protection from EHM [32,76, 77]. In addition, Perkins *et al*. showed a positive correlation between pre-existing TH-1-associated IFN-γ levels in PBMCs and prevention of viremia [71]. In the present study, protection from clinical EHM was associated with a significant and timely upregulation of mRNA expression of pro-inflammatory cytokine and chemokines (IL-1β, CXCL10), TH-1-associated cytokines (IFN-γ, TBET) and a regulatory cytokine (TGF-β) in whole blood of horses following experimental infection.

The upregulation of TBET and IFN-γ mRNA levels in non-EHM horses only in the present study suggests that a TH-1 cellular immune response was associated with protection from EHM and probably resulted in a positive feedback loop that amplified the polarization of the TH-1 pathway [87,89]. While IFN-γ is mainly expressed by natural killer cells (NKs) during innate immune response, there is evidence that equine TH-1 cells and CTLs are the source for IFN-γ during adaptive immunity [88,90]. Our results are also consistent with other studies that show an importance of this type of immunity for protection from herpesviruses in horses and other mammals [33,65, 85, 86, 88, 91,93]

The pro-inflammatory chemokines CCL5 and CXCL10 are important soluble signalling effectors that act through leucocyte recruitment and T-cell activation [45,94]. The expression of CXCL10 is type I IFN dependent [95] and it is therefore not surprising that the mRNA expression profile mirrors the IRF7 and 9 response in the present study. Like IRF7 and IRF9, CXCL10 mRNA expression was significantly increased in blood of non-EHM horses on days 1–3 pi in contrast to EHM horses, which could affect timely and efficient monocyte, NK cell and TH-1 cell recruitment and activation in EHM horses early after infection. However, following day 4 pi with the onset of viremia, CXCL10 mRNA expression was significantly higher in EHM horses compared to non-EHM horses. Additionally, mRNA levels of ‘regulated upon activation normal T-cell expressed and secreted’ (RANTES) and CCL5 were overall higher in EHM horses at baseline compared to non-EHM horses. The second interesting difference is in the fact that CCL5 is modulated in EHM horses but does not change in non-EHM horses. Besides recruiting monocytes, T cells, basophils and eosinophils, CCL5 has been shown to mediate the adhesion of leucocytes to the vascular endothelium as a crucial step for leucocyte migration during inflammation [96]. In mice, CCL5 antagonists have been shown to decrease leucocyte adhesion to the meningeal endothelium during HSV-1 infection [97]. The different mRNA expression profile pre- and post-challenge in EHM horses compared to non-EHM horses indicates a potential role of CCL5 in the pathogenesis of EHM that needs to be evaluated in future studies.

In contrast to the early induction of an IFN response and induction of cellular immunity, we detected significantly increased expression of TGF-β and IL-10 mRNA in EHM horses when compared to blood from non-EHM horses. This upregulation of TGF-β mRNA expression in blood coincided with peak viremia where levels were significantly higher compared to non-EHM horses. High TGF-β mRNA levels during viremia have also been previously reported *in vivo* [33,65] and may contribute to a suppression of EHV-1-specific T-cell responses [98]. Our IL-10 results in blood are consistent with our findings in nasal secretions, where levels of the anti-inflammatory cytokine IL-10 were also significantly higher in EHM horses. Interestingly, EHM horses showed overall significantly higher pre-existing gene expression levels of IL-10 compared to non-EHM horses. An upregulation of IL-10 gene expression during EHV-1 viremia is a common observation [33,44, 65, 67, 72], and can be appreciated in the present study in both groups, but horses that go on to develop EHM showed consistently increased IL-10 when compared to non-EHM horses. IL-10 is discussed in the literature as an important inhibitor of inflammatory responses and T-cell activation [78,99], and is expressed by a broad range of T-cell subsets and by cells of the innate immune system [78,100]. During viral infections the timing of this regulatory mechanism is important to prevent immune-mediated pathology. In human infections, a premature priming of the cells of the innate immune system towards a regulatory phenotype can lead to an uncontrolled immune response that enables unrestricted pathogen dissemination and severe clinical outcome [99,101]. The fact that we observed high pre-existing levels of IL-10 in EHM horses at each of the examined anatomical sites of EHV-1 infection (nasal mucosa, blood, CNS) may be indicative of a shift to a regulatory immune phenotype in EHM horses that consequently leads to a failure to recruit and activate an appropriate TH-1 immune response. This also strengthens the RNA sequencing profile findings by Zarski *et al*. that showed a downregulation of TH-1-associated genes in PBMCs of EHM horses together with a dysregulation of genes associated with T-cell activation [93].

Consistent with a diminished IFN response, a delayed pro-inflammatory cytokine/chemokine response, and high baseline regulatory cytokine levels, are the significantly higher IgG3/5 antibody levels in EHM horses compared to non-EHM horses. High IgG3/5 antibody levels have been linked to a TH-2 biased immune response and have previously been associated with more severe clinical outcome in EHV-1 vaccination studies in horses [63]. IgG antibody isotype ratios have been discussed as potential predictive markers of EHM because horses showing high IgG4/7 levels in relation to IgG3/5 levels appeared to be better protected against neurological disease [63] although this previous study was limited by the small number of horses exhibiting EHM. IgG4/7 antibody secretion is thought to be associated with activation of TH-1 immunity, and in several studies horses showing high IgG4/7 antibody levels appear to be better protected from EHV-1 viral shedding and viremia [71,72]. Confirming these previous findings, we did see some association between total IgG and IgG4/7 pre-infection levels and EHM development. The two old mares with the highest IgG and IgG4/7 pre-infection levels showed only mild EHM and recovered. Furthermore, the two old mares showing the lowest IgG and IgG4/7 pre-infection levels were the first two horses that needed to be euthanized due to severe clinical EHM. However, horses showing antibody levels that would categorize them as being at low risk for EHM development [102] still developed severe fatal EHM in our study. In addition, using the EHV-1 risk evaluation test in samples from a previous study by Holz *et al*., all seven horses were categorized as high-risk for EHV-1 disease. Four of these yearling horses developed respiratory disease but did not show neurological symptoms. The other three horses showed only mild/no respiratory signs but developed EHM and two horses had to be euthanized. This emphasizes that the individual clinical presentation of EHV-1 disease is complex and relying solely on antibody levels as a predictable marker is not advisable in the process of deciding whether to take protective measures such as vaccination or biosecurity.

The immunological phenotype of EHM horses in the present study is probably influenced by the female sex and the age of the horses. In most mammals including horses, the immune system changes with age, which is a process described as ‘immunosenescence’ [39]. While these changes are complex, it has been shown that lymphocytes of elderly humans and horses seem to be impaired in their ability to be activated and proliferate, and a decline in TH-1 cytokine production has been described [103]. In studies using mice it has been suggested that increasing levels of IL-10 in aged individuals may be responsible for the suppression of T-cell activation. This may be due to the upregulation of IL-10-expressing T-regulatory cell subsets during ageing; however, this phenomenon has not been described in horses [39]. In contrast, T-regulatory cells have been shown to be decreased in old horses compared to middle aged horses [39,104]. Interestingly, a decrease of circulating T-regulatory cells in EHM horses during peak viremia was shown for EHM horses of the present study by *in silico* cell sorting as part of our RNA sequencing study [93]. However, FOXP3 expression in the present study remained unchanged in both experimental groups. While the fraction of T-regulatory cell populations decreases with age in the blood circulation, it has been discussed that the percentage of this cell type increases locally in tissues such as the skin of elderly humans [105], and during varicella zoster infection in humans the relevance of tissue resident T-regulatory cells in the skin of the elderly is becoming more apparent. Their suppressive effect on TH-cell activation may contribute to an impaired immune response locally in the skin [105]. The source of the high IL-10 levels in nasal mucosa, PBMCs and CNS of EHM horses in the present study remains to be elucidated and further research is needed to identify local T-cell subsets at each infection site.

Other common age-related underlying endocrine abnormalities may also modify the immune response through changing of the hormonal status [104]. Moreover, the hormonal cycle of the female gender is thought to influence TH cell subset activation systemically and locally [106,107]. A TH-2 biased immune response is described during pregnancy in humans and influenced by high oestrogen and progesterone levels [106]. Furthermore, an upregulation of genes related to the progesterone response and a skew towards a TH-2 cell activation in PBMCs of EHM horses has previously been shown by RNA sequencing analysis [93]. This confirms female sex as a risk factor for EHM [7,32, 108] and aligns with earlier studies that indicate that a hormonally influenced uterine microenvironment during late pregnancy may be advantageous for EHV-1 infection of uterine endothelial cells [40,41].

Endothelial cell infection at the vasculature of the uterus and CNS is central to the pathogenesis of secondary disease manifestations as it leads to inflammation, thrombosis and haemorrhage [6,21]. Inflammatory processes activate proteases that disrupt the basal lamina around vessels, which may lead to necrosis [109]. MMP9 is involved in extracellular matrix remodelling processes, becomes activated during inflammation and degrades the matrix surrounding vascular endothelial cells [109,110]. Interestingly, we detected elevated MMP9 mRNA baseline levels in whole blood of EHM horses that remained significantly higher compared to non-EHM horses until peak viremia. An upregulation of MMP9 has also been shown to be correlated with early stages of ischaemia in human stroke patients and predicts haemorrhagic complications [109,112]. Finally, elevated MMP9 levels have been described during inflammatory processes in the endometrium of mares [113].

Activation of the coagulation cascade *in vitro* [114,117] and *in vivo* during viremia and activation of equine platelets also occur during EHV-1 infection [118]. However, the pathogenesis of thrombosis and ischaemic tissue degeneration during EHV-1 infection is poorly understood. The multifunctional protein THBS1 serves as marker of platelet activation, contributes to haemostasis, and is also located in the vascular vessel wall [119]. Interestingly, we found THBS1 mRNA levels in whole blood to be downregulated during viremia in EHM. THBS1 is known to be involved in inflammatory processes and thrombosis but also in a variety of other mechanisms including cell–cell interaction, and in wound healing [119]. Because of its multipotent character, studies on the role of THBS1 in human ischaemic stroke have been inconclusive when trying to define a precise role within the contribution to thrombosis [119,121]. The potential contribution of MMP9 and THBS1 (dys-)regulation to thrombosis, ischaemia and haemorrhagic infarction leading to EHM/abortion remains speculative but may be worth investigating by further research.

In summary, the current study validates the ‘old mare model’ as an experimental neurological model of EHV-1 infection. Comparing the immune response of EHM horses to horses that were ‘protected’ from EHM (non-EHM horses), our main findings suggest that a protective immunological profile is associated with an early and strong type 1 IFN induction at the nasal mucosa and in the blood as well as a timely upregulation of proinflammatory cytokines and chemokines (IL-1, TBET, CXCL10). This profile is consistent with a TH-1 cellular immune response. In contrast, the immunological phenotype of horses that developed EHM in the present study seems to be shifted toward a regulatory or TH-2 type immune response, as it is characterized by the lack of a type I IFN response at the respiratory tract, a delayed upregulation of type 1 IFN and proinflammatory cytokine/chemokine mRNA in blood, and an overall high proportion of regulatory cytokines (IL-10, TGF-β) as well as high levels of IgG3/5 antibody sub-isotypes. Of note is that due to the nature of our neurological model, the ‘at-risk profile for EHM’ might be biased towards the immunological profile of an aged female horse. However, when comparing the outcomes of the current study with results of three yearling horses that exhibited EHM in a previous study [33], we find that the immunophenotype of these yearling horses mirrors the phenotype described in the current study for horses affected by EHM. Combining the findings of studies that included young horses with EHM with our present data, we suggest that a future vaccine candidate must counteract the ‘at-risk’ immunological profile identified in old female horses to protect horses of all ages from EHM.

## Abbreviations

CNS: central nervous system
CSF: cerebrospinal fluid
dpi: days post-infection
EHM: equine herpesvirus myeloencephalopathy
EHV: equine herpesvirus
hpi: hours post-infection
IgG: immunoglobulin G
IL: interleukin
INF: interferon
IRF: interferon regulatory factor
MFI: mean fluorescent intensity
PBMC: peripheral blood mononuclear cell
PFU: plaque forming unit
pi: post-infection
RANTES: regulated upon activation normal T-cell expressed and secreted
RQ: relative quantity
RT-qPCR: real-time quantitative PCR
TH: t-helper
VN: virus neutralization
